# Three-year follow-up of serum 25-hydroxyvitamin D, parathyroid hormone, and bone mineral density in nursing home residents who had received 12 months of daily bread fortification with 125 μg of vitamin D_3_

**DOI:** 10.1186/1475-2891-12-137

**Published:** 2013-10-11

**Authors:** Veronica Mocanu, Reinhold Vieth

**Affiliations:** 1 Department of Pathophysiology, Grigore T. Popa University of Medicine and Pharmacy, 16 Universitatii str, Iasi 700115, Romania; 2 Department of Laboratory Medicine and Pathobiology, University of Toronto, Toronto, VM, Canada; 3 Department of Nutritional Sciences, University of Toronto, Toronto, RV, Canada

**Keywords:** Geriatrics, Cholecalciferol, Osteoporosis, Vitamin D deficiency, Bread fortification

## Abstract

**Background:**

We conducted a single-arm clinical trial in institutionalized seniors, on the effects of high-dose vitamin D3-fortified bread daily intake (clinicaltrials.gov registration NCT00789503).

**Methods:**

At 1 and 3 years after the dietary fortification was stopped, serum 25-hydroxyvitamin D (25(OH)D), parathyroid hormone (PTH) and bone mineral density were measured in 23 of the original study subjects, aged 60-82 years who had consumed bread buns (100 g) fortified with 320 mg elemental calcium and 125 μg (5,000 IU) vitamin D3 daily for one year.

**Results:**

At the end of the 1-year supplementation phase (receiving vitamin D3 fortified bread daily), mean (SD) serum 25(OH)D was 127.3 ± 37.8 nmol/L (baseline for this follow-up). At 1-year follow-up, the serum 25(OH)D was 64.9 ± 24.8 nmol/L (p = 0.001, vs. baseline); and at 3-year follow-up it was 28.0 ± 15.0 nmol/L (p = 0.001 vs. baseline). Serum PTH was 18.8 ± 15.6 pg/ml at baseline while at Year 3 it was 48.4 ± 18.4 pg/ml (p = 0.001 vs. baseline). Lumbar spine BMD did not change from baseline to Year 3. However, by Year 3, hip BMD had decreased (0.927 ± 0.130 g/cm^2^ vs. 0.907 ± 0.121 g/cm^2^, p = 0.024).

**Conclusion:**

Vitamin D nutritional status exhibits a long half-life in the body, and a true steady-state plateau may not even be reached 1 year after a discontinuation in dose. Furthermore, once the need for vitamin D has been established, based on a low baseline serum 25(OH)D concentrations, the appropriate action is to maintain corrective vitamin D supplementation over the long term.

**Trial registration:**

Clinical trial registration number: NCT00789503

## Background

Older adults are particularly susceptible to vitamin D insufficiency [1] particularly in nursing homes [2]. In Romania, serum 25-hydroxyvitamin D (25(OH)D) concentrations in institutionalized seniors were 28.5 ± 10.8 nmol/L [3], far below the minimum 50 nmol/L that the US Institutes of Medicine has set as the basis for its latest recommended dietary allowance (RDA) [4].

The serum 25(OH)D concentrations likely required to minimize the risk of falls and fractures has been estimated to be between 75 and 100 nmol/l [5]. We previously provided evidence to support this recommendation by completing a single-arm clinical trial in which we provided institutionalized seniors with bread fortified each day with 125 μg (5,000 IU) of vitamin D_3_[3]. This dietary protocol ensured that virtually all of the adults achieved serum 25(OH)D concentrations of at least 75 nmol/L. Here we report the long-term follow-up of study subjects at 1 and 3 years after discontinuing the clinical trial. During this time, the subjects have returned to their previous standard of health care, which did not provide a vitamin D supplement.

## Subjects and methods

### Study population and design

The single-arm clinical trial (clinicaltrials.gov registration NCT00789503) initially enrolled 45 patients (28 women and 17 men), 58 to 89 years of age (71 ± 6.9 years), all residing in a nursing home located in Iasi, Romania (Latitude 47°N) [3]. At the end of the one-year supplementation protocol (consumption of bread buns fortified with 5,000 IU vitamin D_3_ per daily serving) [3], 33 patients were assessed by Dual-energy X-ray absorptiometry (DXA) (January 2005). Initial and final bone mineral density (BMD) data were available for 33 subjects. These patients were retested by DXA one and three years after the vitamin D protocol was discontinued (January 2006 and January 2008, respectively).

### Study protocol

In the original study, all subjects received 125 μg (5,000 IU) vitamin D_3_/day and 800 mg calcium carbonate/day (320 mg of elemental calcium) for 1 yr.

Bone mineral density (BMD) of the lumbar spine and proximal femur was measured at baseline and after 12 months of vitamin D_3_ supplementation [3]. The BMD measurements at the end of vitamin D_3_ supplementation were taken as the baseline values for this follow-up report (i.e. “baseline” refers to measures obtained at the end of 1 yr of vitamin D), and additional measurements were obtained after 12 and 36 months of follow-up study. Blood for serum calcium, 25(OH)D and serum PTH was obtained at baseline and at 1 and 3 years follow-up.

At the beginning of the original study, the 10-day food records obtained from the nursing home showed that the per-day vitamin D intake was 84 IU, while calcium intake was 717 mg. The participants did not take supplements of calcium, vitamin D, or both in the preceding 36 mo.

Both the intervention and follow-up studies were approved by the Human Investigation Review Committee at Grigore T. Popa University, and written informed consent was obtained from each subject.

### Blood collection and analytical methods

Fasting blood samples were collected at baseline and after 1 and 3 years of follow-up study. Serum and plasma were kept frozen until analysis.

Serum calcium concentrations were measured by standard laboratory methods. Serum 25(OH)D concentrations were assayed by HPLC method [6] (results were validated through the DEQAS proficiency survey (http://www.deqas.org) for 25(OH)D, and the results were within the all-laboratory methods mean). Serum intact PTH was measured by an enzyme-amplified “two-step” sandwich-type immunoassay (DSL, Webster, Texas, USA) with an inter-assay CV ranging from 6.0 to 6.3% (normal range for adults 40-70 years of age, pg/ml). The BMD of the lumbar spine, femoral neck and trochanter were measured by dual energy x-ray absorbtiometry (Hologic, Delfi A and Delfi W). The calibration of bone densitometer was performed by scanning the European Spine Phantom. All measurements were performed by the same experienced technician.

### Statistical analysis

Statistical analyses were performed using SPSS version 15.0 (SPSS Inc, Chicago). Data are expressed as mean ± SD. The distributions of all variables were tested with Kolmogorov-Smirnov tests. Descriptive statistics (mean and SD) were determined for all variables. Effects of the follow-up after the vitamin D protocol was completed were examined using the paired t-test versus the “baseline” values for each subject, which in the context of the present follow-up report are those values obtained at the end of the 1-yr intervention with vitamin D-fortified bread. A two-way repeated-measures ANOVA was conducted to explore the impact of sex and age on outcome variables (lumbar and hip BMD, concentrations of serum calcium, 25(OH)D, and PTH).

## Results

### Characteristics of the population

Thirty-three older nursing home residents were included into the follow-up study, which represents the population of participants having completed the Vitamin D fortified bread intervention. Baseline characteristics of the patients are shown in Table 1. Two patients withdrew from the study before the 12 month-follow-up visit. One patient died, and other 7 withdrew by 3-year follow-up. Complete, 36-month follow-up data were available for 23 subjects. Twenty-three patients had initial and final of BMD data by 36 month follow-up study.

**Table 1:** **Baseline characteristics of the patients having received 125 μg (5,000 IU) vitamin D**_**3**_**/day and 800 mg calcium carbonate/day (320 mg of elemental calcium) for 12 months (N = 23)**

**Characteristic**	**Males**	**Females**	**Total**
Number	11 (33%)	22 (66.6%)	33 (100%)
Age (yr)	67.0 ± 5.2	69.1 ± 5.1	68.3 ± 5.6
Weight (kg)	81.5 ± 11.9	71.2 ± 10.2	76.3 ± 11.3
Height (cm)	167.0 ± 4.3	154.0 ± 5.5	159.4 ± 7.3
BMI (kg/cm^2^)	29.3 ± 4.6	30.5 ± 4.2	30.0 ± 4.3
Vitamin D intake (μg/d)	2.1 ± 1.1	1.5 ± 0.9	1.7 ± 1.0
Calcium intake (mg/d)	938 ± 432	758 ± 341	780 ± 387

Data expressed in mean ± SD.

### Measurements of serum 25-hydroxyvitamin D

At baseline for the original clinical trial, mean serum 25(OH)D was 28.5 ±10.8 nmol/L and, after 12 months of 5,000 IU/d vitamin D_3_ bread supplementation, this increased to 125.6 ±38.8 nmol/L [3]. Twelve months after the end of the clinical trial, mean serum 25(OH)D was 64.9 ± 24.8 nmol/L, a significant decline from its peak value (p = 0.0001) (Table 2).; however, serum 25(OH)D concentrations exceeded 50 nmol/L in 65% of the patients (15 of 23 patients). However, at 36 month follow-up mean serum 25(OH)D has essentially returned to the baseline value of the original clinical trial (28.0 ± 15.0 nmol/L).

**Table 2:** Biochemical values and BMD changes before vitamin D bread consumption, at baseline of follow-up study (after 12 mo of vitamin D bread consumption) and after 12 and 36 months the supplementation with vitamin D was discontinued

**Parameter**	**Vitamin D bread consumption (-12 mo)**	**Baseline of follow-up study (0 month)**	**12 month follow-up**	**36-month follow-up**	**P-value**^**1**^	**P-value**^**2**^	**P-value**^**3**^
Serum calcium (Normal: 2.15-2.55 mmol/L)	2.30 ± 0.14	2.31 ± 0.13	2.26 ± 0.13	2.29 ± 0.15	0.18	0.49	0.07
Serum 25(OH)D (Sufficiency: 50-125 nmol/l)	29.8 ± 9.3	127.3 ± 37.8	64.9 ± 24.8	28.0 ± 15.0	<0.001	<0.001	<0.001
Serum PTH (Normal: 16-62 pg/ml)	60.2 ± 42.6	18.8 ± 15.6	46.7 ± 21.2	48.4 ± 18.4	<0.001	<0.001	<0.001
Lumbar spine BMD (g/cm2)	0.825 ± 0.113	0.858 ± 0.134	0.855 ± 0.146	0.867 ± 0.142	0.81	0.32	0.65
Total hip BMD (g/cm2)	0.736 ± 0.128	0.927 ± 0.130	0.913 ± 0.130	0.907 ± 0.121	0.02	0.02	0.14

N = 23, Data expressed in mean ± SD.

^1^Student’s *t* test for paired data (baseline vs. 12 month follow-up).

^2^Student’s t test for paired data (baseline vs. 36 month follow-up).

^3^Repeated-measures ANOVA. The interaction effect between sex and age was not statistically significant for any variable. There was a statistically significant main effect for age on 25(OH)D (F = 4.69, p = 0.007, partial eta squared = 0.92) and for gender on total hip BMD (F = 7.08, p = 0.05, partial eta squared = 0.59).

### Measurements of serum calcium and PTH

There were no significant differences in serum calcium concentrations at baseline versus 12 and 36 month follow-up (Table 2). Compared to the end-of clinical trial, PTH concentrations significantly increased by 12 months of follow-up (p = 0.0001) and by 36 months (p = 0.0001) but remained within the reference range. No treatment-related adverse events were observed during follow-up.

### Bone mineral density measurements

Upon withdrawal of vitamin D_3_ (125 μg) supplementation, there were no significant differences in the mean percentages of change in lumbar spine; the mean percentages of change from baseline in lumbar spine BMD at 1-year follow-up was –0.31% and at 3-year follow-up was 1.03%. Statistically significant changes from baseline were noticed in hip BMD (Table 2); the mean relative change from baseline in hip BMD at 1-year follow-up was -1.52% and at 3-year follow-up was -1.99%.

## Discussion

Corrective action was not implemented after the original clinical trial was completed because the study subjects returned to their standard of care. Until the end of this follow-up, there was no compelling reason to provide more vitamin D. Although it was reassuring that at follow-up Year 1, the serum 25(OH)D was appropriate for the group, the data at follow-up Year 3 show that 25(OH)D had not established its new plateau. Previous studies suggests that when vitamin D supplementation is stopped, the serum 25(OH)D concentrations exhibit an apparent half-life of two months [7]. However, the decline in serum 25(OH)D observed here exceeded 1 year (i.e. the decline exceeded six of the presumptive 2-month half-life); therefore, the apparent half life can be substantially longer than what has been previously estimated. One reason for a longer apparent half-life of the decline in serum 25(OH)D, compared to a tracer 25(OH)D compound, is because firstly, unmetabolised vitamin D is released from tissue stores built up during vitamin D supplementation, and secondly, there is a background of newly acquired vitamin D from the environment or diet that continues to be generate 25(OH)D.

At one year after the end of the clinical trial, the group as a whole met the 50 nmol/L criterion for serum 25(OH)D as recently established by US Institute of Medicine. While this was apparently reassuring, the further decline in 25(OH)D shown here emphasizes the need for sustained awareness and monitoring of vitamin D status in nursing home residents. Follow-up reports after discontinuation of vitamin D and calcium supplementation are sparse. One report of a similar population that was provided lower doses of vitamin D, showed that bone turnover markers had returned to baseline by two years after discontinuation, but serum 25(OH)D concentrations were not reported [8].

The present report is to our knowledge the longest follow-up after discontinuation of vitamin D, and it does show a sustained biochemical benefit to the one-year point, in that the serum 25(OH)D exceeded 50 nmol/L. However, by follow-up Year 3, the benefits of vitamin D fortification, were essentially gone. At follow-up Year 1, PTH had increased significantly from the end-of-vitamin D baseline. At follow-up Year 3, there was evidence that bone loss had progressed at the hip, but since there was no reference group in this study, the bone loss may reflect the normal age-related decline. The strength of this study is in its 3-yr characterization of the progression of the decline in 25(OH)D in older adults who had before-hand, received enough vitamin D to achieve 25(OH)D concentrations that most would consider to be in the optimal range. Among the weaknesses of this work is the lack of a reference group against which to compare the variables studied.

## Conclusions

In retrospect, it is unfortunate that the serum 25(OH)D concentrations eventually returned to the osteomalacic levels present before the subjects initially entered the bread-fortification clinical trial. Lessons learned here are, firstly, that vitamin D nutritional status exhibits a long half-life in the body, whereby follow-up testing even at 1 year after a change in dose may not reflect a true plateau (i.e. steady-state) value; and secondly, that when a need for nutrient is established, even if this recognition is as part of a research protocol, then the appropriate action is to institute corrective measures beyond the extent of the research protocol.

## Competing interests

The authors declare that they have no conflict of interests.

## Authors’ contributions

The authors’ responsibilities were as follows: VM principal investigator, study concept and design, acquisition of data, analysis and interpretation of the data, drafting of the manuscript; RV study concept and design, analysis and interpretation of the data; critical revision of the manuscript. Both authors read and approved the final manuscript.
